# Hydrogel-assisted deficit irrigation improves sugar beet yield, quality, and water-use efficiency in arid sandy loam soils

**DOI:** 10.3389/fpls.2026.1913358

**Published:** 2026-08-24

**Authors:** Lamy Hamed, Eman I. R. Emara

**Affiliations:** 1 Department of Environment and Agricultural Natural Resources, College of Agricultural and Food Sciences, King Faisal University, Al-Ahsa, Saudi Arabia; 2 Department of Agronomy, Faculty of Agriculture, Cairo University, Giza, Egypt

**Keywords:** *Beta vulgaris* L., drip irrigation, drought, superabsorbent polymer, water productivity

## Abstract

**Introduction:**

Water scarcity is a major constraint to sugar beet production in sandy soils due to low water-holding capacity and rapid drainage.

**Methods:**

A two-season field experiment was conducted to evaluate the combined effects of deficit irrigation and hydrogel application on sugar beet yield, juice quality, and water-use efficiency (WUE). Four irrigation regimes were tested: I1 (100% ETc), I2 (85% ETc), I3 (70% ETc), and I4 (55% ETc), in combination with four hydrogel rates: H0 (0), H1 (5), H2 (10), and H3 (15 kg ha^−1^).

**Results and discussion:**

Deficit irrigation significantly increased sucrose concentration and WUE, but reduced root yield from 101.37-103.55 to 80.09-81.73 t ha^-1^ under sever water deficit (55% Etc).Hydrogel application significantly enhanced root yield (88.12-96.39 t ha^-1^), sugar yield (12.65-15.52 t ha^-1^), sucrose content, and WUE while reducing juice impurities. Although the irrigation × hydrogel interaction was not statistically significant (*P* > 0.05), the combination of moderate deficit irrigation (85% ETc) with 10–15 kg ha^−1^ hydrogel consistently provided the most favorable balance between productivity, water savings, and economic performance. In contrast, full irrigation combined with 15 kg ha^−1^ hydrogel achieved the highest net return, whereas moderate deficit irrigation without hydrogel produced the greatest benefit-cost ratio.

## 1 Introduction

Water scarcity is increasingly recognized as one of the most critical constraints limiting agricultural productivity, particularly in arid and semi-arid regions where irrigation is essential for crop production. Under these conditions, improving water productivity rather than maximizing yield per unit area has become a central objective of sustainable agriculture (Ferreira et al., 2024; Abdelfattah and Mostafa, 2024; Emara et al., 2026). Deficit irrigation (DI) has therefore gained considerable attention as a water-saving strategy that can enhance water use efficiency (WUE) while maintaining acceptable yields. Nevertheless, its effectiveness is strongly influenced by soil characteristics, especially in coarse-textured soils, where rapid water loss and low water-holding capacity reduce its efficiency (Agbna and Zaidi, 2025).

Although deficit irrigation has been extensively investigated as a water-saving strategy and hydrogels have demonstrated considerable potential for mitigating drought stress, limited information is available on their combined application under field conditions for sugar beet production. In particular, the interactive effects of different deficit irrigation levels and hydrogel application rates on crop productivity, juice quality, water-use efficiency, and economic performance under arid conditions remain insufficiently explored. Addressing this knowledge gap is essential for developing integrated and sustainable irrigation management strategies for sugar beet cultivation.

Sugar beet (*Beta vulgaris* L.) is one of the world’s principal sugar crops, contributing approximately 20% of global sugar production (Soare et al., 2021). In Egypt, it has become the predominant source of domestically produced sugar, accounting for more than 62% of national sugar output, with a cultivated area exceeding 286,000 ha and an annual root production of approximately 14.2 million tons (Abdelwahab et al., 2022; Ramadan et al., 2025). Although sugar beet is relatively tolerant to moderate water deficit, adequate water supply remains essential during critical developmental stages to maintain root growth, sucrose accumulation, and technological quality. Therefore, efficient irrigation management is particularly important for sustaining sugar beet productivity under arid and semi-arid conditions. Water deficit conditions can adversely affect root development, sucrose accumulation, and sugar recovery, ultimately reducing both yield and economic returns (Rai et al., 2023; Ismail et al., 2025; Emara et al., 2026). Consequently, optimizing irrigation management for sugar beet under limited water availability remains a priority for enhancing productivity and sustainability.

In parallel, recent advances in soil amendment technologies have highlighted hydrogels, also known as a superabsorbent polymers (SPAs), as promising materials for mitigating drought stress and enhancing crop performance under water-limited conditions. Hydrogel absorb and store large quantities of irrigation water and gradually release it into the root zone as the surrounding soil dries, thereby improving soil water availability, reducing irrigation frequency, and alleviating drought stress (Abdelghafar et al., 2024; Rodrigo and Munaweera, 2025). In addition, to improving water availability, hydrogels reduces nutrient losses through leaching, enhance nutrient retention, promote root growth, and improve water use efficiency. These benefits are particularly pronounced in sandy and sandy loam soils, where low organic matter content and limited water-holding capacity restrict crop productivity (Rai et al., 2023). Consequently, integrating hydrogels with deficit irrigation has considerable potential to improve crop productivity while maximizing the efficiency of limited irrigation water under arid conditions.

Although both deficit irrigation and hydrogel application have been extensively studied, most research has treated these approaches independently, resulting in fragmented understanding of their combined potential. Emerging evidence suggests that integrating hydrogels with deficit irrigation could create a synergistic effect by stabilizing soil moisture dynamics and improving plant water uptake efficiency under stress conditions (Patra et al., 2022; Ismail et al., 2025). However, the interaction between irrigation regimes and hydrogel application rates remains insufficiently explored, particularly under field conditions and in sandy soils where such strategies are most needed. Furthermore, recent reviews emphasize that hydrogel-soil-plant interactions are complex and context-dependent, highlighting the lack of comprehensive studies addressing optimal combinations of water management and soil amendments (Agbna and Zaidi, 2025).

Based on the aforementioned considerations, it is hypothesized that the integration of deficit irrigation with hydrogel application will mitigate the adverse effects of water stress by improving soil moisture retention, enhancing plant water uptake, and ultimately sustaining sugar beet yield and quality under reduced irrigation levels. It is further expected that moderate deficit irrigation combined with optimal hydrogel rates will achieve higher water use efficiency without significant yield penalties compared to full irrigation. Therefore, the objectives of this study were to: (i) evaluate the effects of different irrigation regimes on sugar beet, yields, quality attributes, and water use efficiency (WUE); (ii) assess the effectiveness of different hydrogel application rates in improving crop productivity and WUE; (iii) investigate the combined effects of irrigation regimes and hydrogel application on water use efficiency and sugar productivity; and (iv) evaluate the economic feasibility of the proposed irrigation-hydrogel management strategies to identify the most productive, water-efficient, and economically sustainable approach for sugar beet production under water-limited conditions.

## 2 Materials and methods

### 2.1 Experimental site and duration

Field experiments were conducted at a private farm, Alexandia desert road, Egypt (30.1924° N, and 30.8088° E), over three consecutive growing seasons (2022/2023, and 2023/2024). The experimental soil was classified as sandy loam with low organic matter content and limited water-holding capacity. Meteorological data, including temperature, relative humidity, and reference evapotranspiration (ET_0_), were obtained from a nearby weather station following FAO guidelines (Allen et al., 1998). Mean air temperature during winter months (December – February) ranged from 13-19 °C up to 22-34 °C during March-May. Rainfall was negligible and irregular, with total annual precipitation approximately 15–20 mm, during winter months. The region is characterized by low relative humidity and frequent dry winds, particularly during spring.

### 2.2 Experimental design and layout

**Figure 1:**
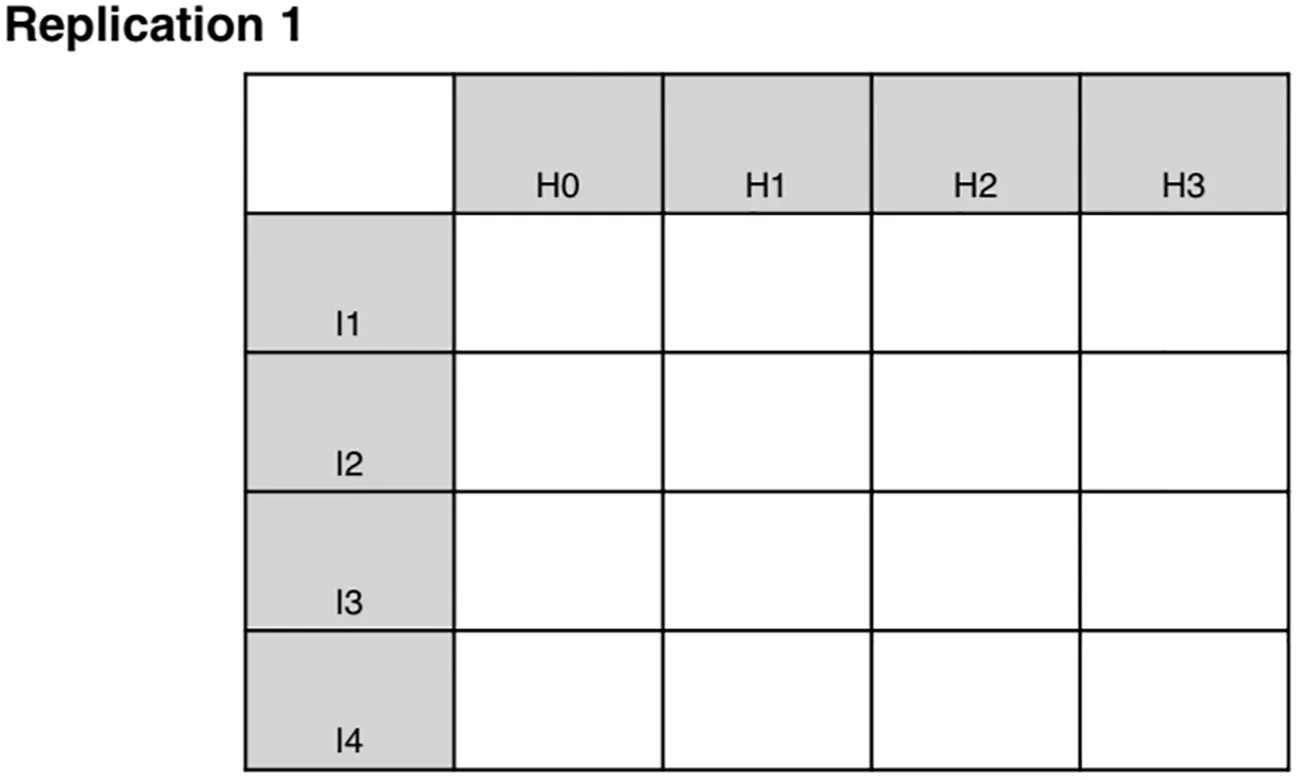
Schematic representation of the strip split-plot experimental design showing irrigation regimes (I1-I4) arranged as vertical strips and hydrogel rates (H0-H3) arranged as horizontal strips within each replication. Replications 2 and 3 follow the same arrangement.

The field experiment was conducted using a strip split-plot design with three replications. Irrigation regimes were assigned to the main vertical strips, whereas hydrogel application rates were allocated to the horizontal subplots within each irrigation strip. Four irrigation regimes (I1, I2, I3, and I4) and four hydrogel application rates (H0, H1, H2, and H3) were evaluated, resulting in 16 treatment combinations per replication and a total of 48 experimental plots. Each irrigation strip comprised four adjacent hydrogel subplots, each measuring 20 m^2^. Buffer zones were established between adjacent irrigation strips to minimize lateral water movement and maintain treatment independence. The perpendicular arrangement of irrigation strips and hydrogel subplots enabled the evaluation of the main effects of both experimental factors and their interaction. A schematic representation of the experimental layout is provided in **Figure 1**.

### 2.3 Soil sampling and physicochemical analysis

Composite soil samples (0–60 cm depth) were collected prior to sowing and analyzed using standard procedures. Soil texture was determined using the hydrometer method (Gee and Bauder, 1986). Soil pH and electrical conductivity (EC) were measured in a 1:2.5 soil-water suspension (Page, 1982). Soil organic matter was determined following the method of Nelson and Sommers (1996). Nitrogen, phosphorus, and potassium were determined using Kjeldahl digestion, Olsen extraction, and flame photometry, respectively (Bremner, 1996; Olsen, 1954). Cation exchange capacity (CEC) was also measured (Sumner and Miller, 1996). Detailed soil properties are presented in **Table 1**.

**Table 1:** Physical and chemical characteristics of the experimental soil before sowing at the study site.

Soil property	Value	Soil property	Value
Sand (%)	78.4	Organic matter (%)	0.46
Silt (%)	11.8	ECe (dS m^−1^)	1.34
Clay (%)	9.8	CaCO_3_ (%)	3.2
Textural class	Sandy Loam	Available N (mg kg^−1^)	37.5
Soil pH (1:2.5)	7.82	Available P (mg kg^−1^)	6.8
Bulk density (Mg m^−3^)	1.58	Available K (mg kg^−1^)	112
Field capacity (%)	14.9	Available water (%)	8.6
Permanent wilting point (%)	6.3	CEC (cmol (+) kg^−1^)	6.9

### 2.4 Experimental treatments

#### 2.4.1 Irrigation regimes

Irrigation treatments were based on crop evapotranspiration (ETc), calculated using the FAO Penman-Monteith equation (Allen et al., 1998): I_1_ = 100% Etc, I_2_ = 85% Etc, I_3_ = 70% Etc, and I_4_ = 55% ETc.


$$\text{ETc}={\text{ET}}_{0}\times \text{Kc}$$

where ETc is crop evapotranspiration (mm day^-1^), where ET_0_ is the reference evapotranspiration (mm day^-1^) and Kc is the crop coefficient for sugar beet. Irrigation treatments were established by applying 100, 85, 70, and 55% of ETc, corresponding to treatments I1, I2, I3, and I4, respectively, and Kc is the crop coefficient corresponding to the sugar beet growth stage (Initial from 0–30 days: 0.35; Development from 31-70: 0.75; Mid-season from 71-140: 1.15 and Late season from 141- 200: 0.70, Allen et al., 1998).

Irrigation water was applied through a drip irrigation system equipped with inline emitters delivering 4 L h^-1^. The irrigation system was operated at the recommended pressure to ensure uniform water distribution throughout the experimental area). Irrigation scheduling was based on crop water requirements throughout the growing season, and the total volume of irrigation water applied to each treatment was recorded using calibrated water meters. The seasonal irrigation amounts applied under 7200, 6120, 5040 and 3960 m^3^ ha^-1^ in the first season and 7000, 5950, 4900 and 3850 m^3^ ha^-1^ in the second season for I1, I2, I3, and I4 treatment, respectively.

#### 2.4.2 Hydrogel application

A potassium polyacrylate-based hydrogel was applied at rates of 0, 5, 10, and 15 kg ha^−1^, based on the manufacturer’s recommended application range for agricultural use in sandy soils and on rates commonly adopted in previous studies evaluating superabsorbent polymers under water-limited conditions (Abedi-Koupai et al., 2008; Watanabe et al., 2019; Abdelghafar et al., 2024). These rates represent low, intermediate, and relatively high application levels, enabling the evaluation of the dose-dependent response of sugar beet to hydrogel application under different irrigation regimes while ensuring both agronomic relevance and practical applicability under field conditions.

### 2.5 Plant material and agronomic practices

Sugar beet (*Beta vulgaris* L., var. BTS 645) was used in this study. Sowing was carried out in late September using precision drilling, with 50 cm row spacing and 20 cm plant spacing. Seed Rate: 7 kg ha^−1^. Each plot measured 10.5 m^2^ (3.5 × 3 m). Recommended fertilization (240 kg ha^-1^, 70 kg P_2_O_5_ ha^-1^, and 100 kg K_2_O ha^-1^), and crop management practices were uniformly applied. Other agronomic practices were applied uniformly across all treatments (Cooke and Scott, 2012).

### 2.6 Application of hydrogel

The hydrogel used in this study is a polymer composite made by Lucky Star TG., French that contain 40% hydro polymer, 4.8% phosphorus, 6.5% nitrogen, 8.2% potassium, 300-500% storage capacity, and trace of amino acids, cytokinin and various vitamins. Hydrogel was applied once before sowing. It was thoroughly mixed into the soil at a depth of 0–20 cm within the root zone to enhance soil water retention capacity.

### 2.7 Measurements

#### 2.7.1 Quality parameters

At harvesting (200 DAP), a sample of ten roots were taken at random from each sub-plot cleaned and sent to Sugar Beet Laboratory at Nubaria Sugar Factory, El-Beheira Governorate, Egypt, to determine the following:

1. Sucrose percentage: was estimated by using saccharometer lead acetate extract of fresh macerated roots according to Carruthers and Oldfield (2013).
2. Impurities component i.e. alpha amino N, Na, and K (meq 100 g**^-1^** fresh beet) according to the method as described by A.O.A.C methods (AOAC, 2016).
3. Sucrose loss to molasses percentage (SLM) according to Renfield et al. (1993); SLM=(0.343∗(NA+K)+0.94∗(alpha amino N)−0.31)Sugar recovery percentage (SR%): was estimated according to Renfield et al. (1993) by using the following formula:

SR%=pol−[0.343(K+Na)+0.094 α-amino N+0.29].

Where: Pol = sucrose percentage

#### 2.7.2 Yield parameters

Root yield (t ha^−1^) was determined at harvest, and sugar yield was calculated as:


$$\text{Sugar yield}\;({\text{t ha}}^{-1})=\text{Root yield }\left({\text{t ha}}^{-1}\right)\times \text{Sugar recovery\%}$$

#### 2.7.3 Water relations

Total applied water (m^3^ ha^-1^) was determined, as well as Water use efficiency (WUE) was calculated as:


$$\text{WUE}=\frac{\text{Root yield}\left({\text{kg ha}}^{-1}\right)}{\text{Total applied water}\left({\text{m}}^{3}{\text{ ha}}^{-1}\right)}$$

### 2.8 Economic analysis

An economic analysis was conducted to assess the profitability of the different irrigation regimes and hydrogel application rates. Gross return (GR) was estimated by multiplying the root yield by the average market price of sugar beet prevailing during the study period. Total production cost (TC) comprised fixed production costs in addition to irrigation and hydrogel application expenses. Net return (NR) was calculated as the difference between gross return and total production cost, while the benefit-cost ratio (BCR) was determined as the ratio of gross return to total production cost. Economic water productivity (EWP) was calculated by dividing net return by the total volume of irrigation water applied and was expressed as EGP m^-3^.


GR=RY x P

NR=GR−TC

$$BCR=\frac{GR}{TC}$$

$$EWP=\frac{NR}{WA}$$

The official procurement price of sugar beet roots (1,200 EGP t^-1^) was based on the government-announced procurement price for the study period. The hydrogel cost was determined from the commercial purchase price paid during the experiment, while irrigation and other production costs were calculated from the actual production records maintained throughout the experimental period. Economic analysis was conducted following the standard partial budget analysis procedures described by CIMMYT (1988).

### 2.9 Statistical analysis

Data for all measured variables were subjected to analysis of variance (ANOVA) as a strip split plot design (Gomez and Gomez, 1984). Normality and homogeneity of variance were tested using Shapiro Wilk and Levene tests, respectively. To assess the consistency of treatment responses across growing seasons, a combined ANOVA was performed with Year considered as a fixed factor, while irrigation regime (I), hydrogel application rate (H), and their interaction (I × H) were included as fixed treatment effects. When significant differences were detected, treatment means were separated using the least significant difference (LSD) test at the 5% probability level. As the interaction between irrigation regime and hydrogel application represented a principal objective of the study, the corresponding ANOVA results, including F-values, P-values, and interaction effects, are presented and discussed in detail for the key agronomic indicators, namely root yield, sugar yield, and water-use efficiency.

## 3 Results and discussion

### 3.1 Juice quality responses to irrigation regimes

**Figure 2:**
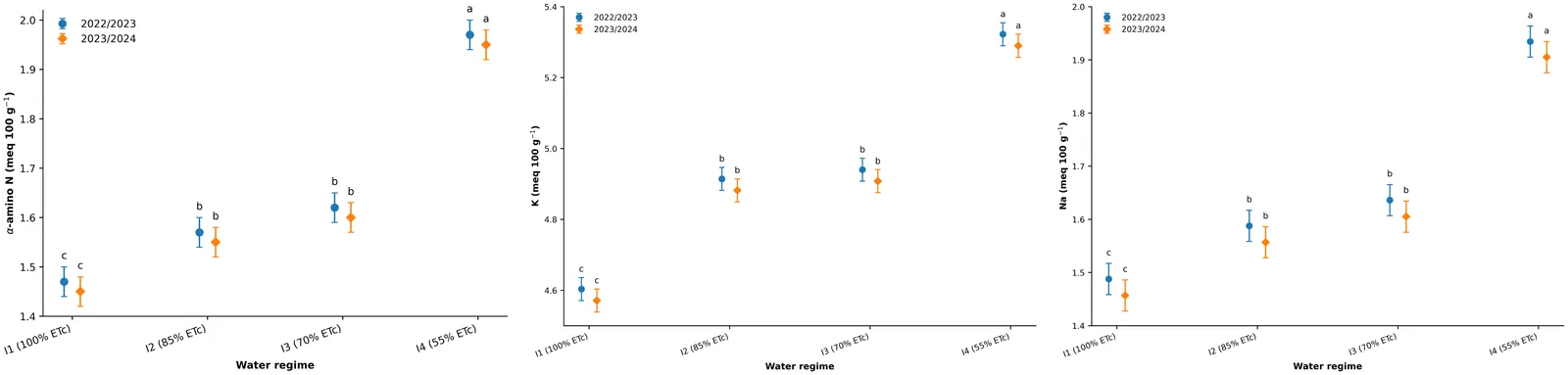
Effect of irrigation regimes on juice impurities (Na, K, and α-amino N) of sugar beet during both seasons. Bars represent mean ± SE. Different letters indicate significant differences at *P* ≤ 0.05.

**Figure 3:**
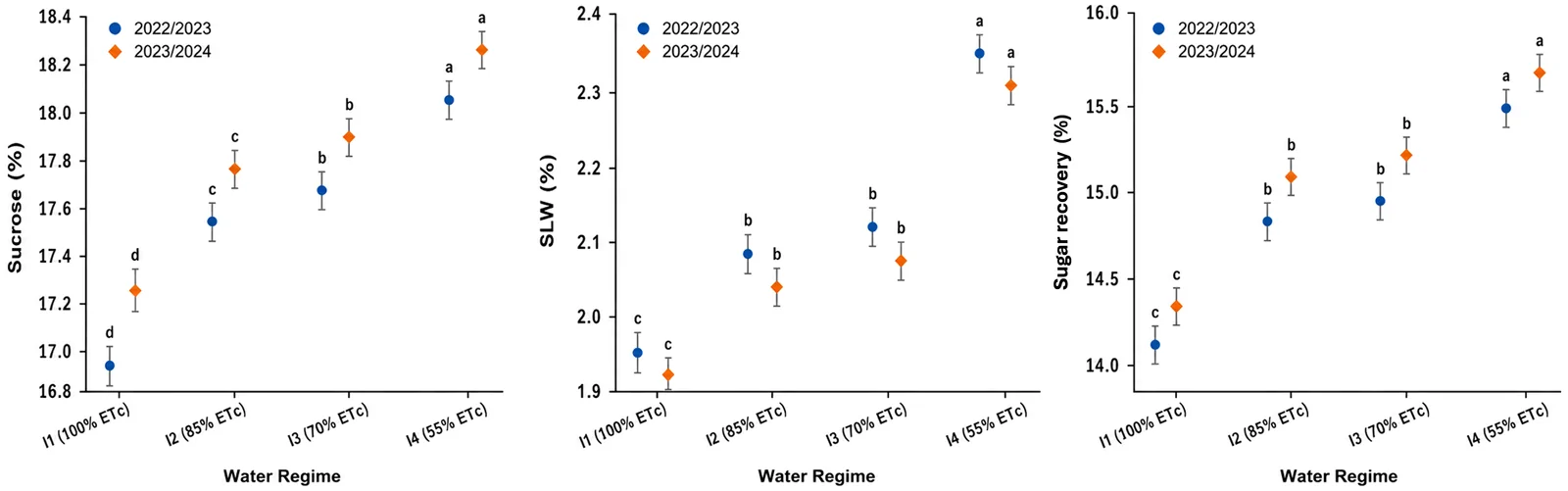
Effect of irrigation regimes on sucrose, Sucrose loss to molasses (SLM), and sugar recovery of sugar beet during the 2022/2023 and 2023/2024 seasons. Bars represent mean ± SE. Different letters indicate significant differences at *P* ≤ 0.05.

Irrigation regime significantly affected all measured quality attributes in both growing seasons (**Figures 2**, **3**). Progressive reduction in irrigation from I1 (100% ETc) to I4 (55% ETc) increased sucrose concentration from 16.89 to 18.01% in 2022/2023 and from 17.19 to 18.31% in 2023/2024. Likewise, sugar recovery increased from 13.92 to 15.49% and from 14.25 to 15.82% across the same treatments, respectively. These findings indicate that water deficit enhanced concentration-based quality parameters, likely through reduced root water content and increased dry matter accumulation.

However, severe water restriction also increased technological impurities. Sodium, potassium, α-amino N, and sucrose loss to molasses (SLM) were consistently highest under I4. For example, Na increased from 1.48 to 1.93 meq 100 g^-1^ in the first season, while SLM rose from 1.93 to 2.37%. Similar trends were observed in the second season. This suggests that although drought stress increased sucrose percentage, it simultaneously impaired juice purity and processing efficiency.

The dual response observed here is well documented in sugar beet physiology. Moderate dehydration often concentrates sucrose in storage roots, whereas prolonged stress disrupts nutrient balance, increases osmolyte accumulation, and elevates molasses-forming constituents that reduce extractable sugar. Recent studies reported similar increases in sucrose concentration under deficit irrigation, but emphasized that high impurity content can offset industrial benefits (Abdelfattah and Mostafa, 2024; Ismail et al., 2025). Broader drought analyses likewise indicate that water stress frequently improves soluble solids concentration while reducing overall technological quality when stress becomes severe (Seleiman et al., 2021).

Therefore, the present findings demonstrate that irrigation deficit improved apparent quality, but severe stress reduced processing quality through impurity accumulation. From a commercial standpoint, this distinction is critical because factory profitability depends on recoverable sugar rather than sucrose concentration alone.

### 3.2 Juice quality responses to hydrogel application

**Figure 4:**
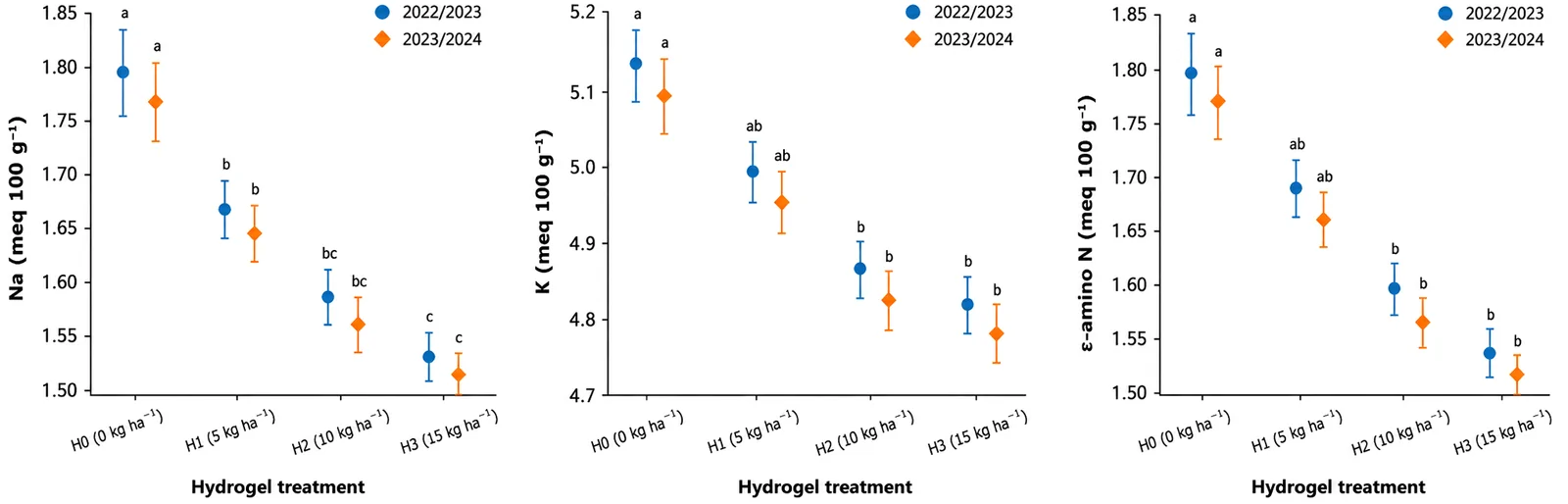
Effect of hydrogel application rates on juice impurities (Na, K, and α-amino N) of sugar beet during both seasons. Bars represent mean ± SE. Different letters indicate significant differences at *P* ≤ 0.05.

**Figure 5:**
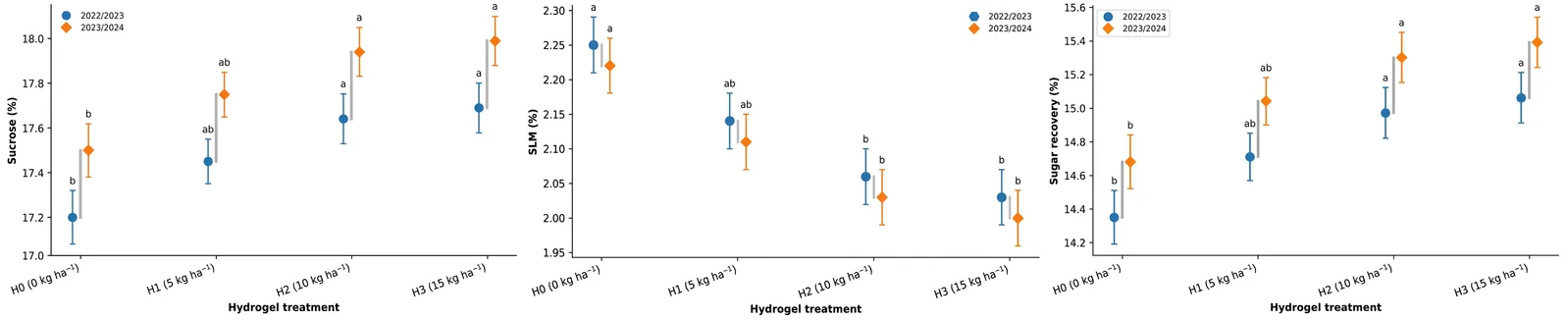
Effect of hydrogel application rates on sucrose, Sucrose loss to molasses (SLM), and sugar recovery of sugar beet during the 2022/2023 and 2023/2024 seasons. Bars represent mean ± SE. Different letters indicate significant differences at *P* ≤ 0.05.

Hydrogel application significantly improved juice quality and reduced impurity levels in both seasons (**Figures 4**, **5**). Increasing hydrogel rate from H0 to H3 raised sucrose concentration from 17.20 to 17.69% in 2022/2023 and from 17.50 to 17.99% in 2023/2024. Sugar recovery also increased from 14.35 to 15.06% and from 14.68 to 15.39%, respectively.

In contrast, Na, K, α-amino N, and SLM declined progressively with hydrogel addition. For instance, Na decreased from 1.80 to 1.54 meq 100 g^−1^ in the first season, while SLM declined from 2.25 to 2.03%. Similar reductions occurred in the second season. These improvements indicate that hydrogel created a more favorable root-zone environment by buffering soil moisture fluctuations and reducing physiological stress.

Hydrogels are known to increase soil water-holding capacity, reduce evaporation losses, and improve nutrient retention, especially in coarse-textured soils. Under such conditions, plants experience less transient water stress and maintain more balanced metabolism, leading to higher sucrose accumulation and lower impurity formation. These mechanisms are supported by recent field and review studies demonstrating that superabsorbent polymers enhance crop quality under drought-prone environments (Abdelghafar et al., 2024; Bhuyan and Jeong, 2025; Hajirad et al., 2026).

The relatively small difference between H2 and H3 for several variables suggests diminishing returns at higher hydrogel rates. Thus, 10 kg ha^−1^ may represent a biologically efficient rate under the current sandy-soil conditions, while higher doses may provide only marginal additional benefit.

### 3.3 Root yield and sugar yield responses to irrigation regimes

Yield traits were strongly influenced by irrigation level (**Table 2**). Root yield decreased significantly with increasing water deficit, falling from 101.37 and 103.55 t ha^−1^ under I1 to 80.09 and 81.73 t ha^−1^ under I4 in the first and second seasons, respectively. This corresponds to yield reductions of approximately 21 and 22% relative to full irrigation.

**Table 2:** Effect of irrigation regimes on root and sugar yields of sugar beet during the 2022/2023 and 2023/2024 seasons.

Treatment	2022/2023		2023/2024	
	Root yield (t ha^−1^)	Sugar yield (t ha^−1^)	Root yield (t ha^−1^)	Sugar yield (t ha^−1^)
I1 (100% ET_c_)	101.37 ± 1.29 ^a^	14.11 ± 0.27 ^a^	103.55 ± 1.32 ^a^	14.76 ± 0.29 ^b^
I2 (85% ET_c_)	97.42 ± 1.07 ^b^	14.38 ± 0.21 ^a^	99.51 ± 1.10 ^b^	15.02 ± 0.22 ^a^
I3 (70% ET_c_)	92.98 ± 1.27 ^c^	13.88 ± 0.24 ^b^	94.98 ± 1.30 ^c^	14.48 ± 0.25 ^b^
I4 (55% ET_c_)	80.09 ± 0.91 ^d^	12.04 ± 0.18 ^c^	81.73 ± 0.93 ^d^	12.93 ± 0.19 ^c^

Data represent mean ± SE. Different letters indicate significant differences at *P* ≤ 0.05.

Sugar yield followed a similar pattern, although the highest value in 2023/2024 was recorded under I2 (15.02 t ha^−1^), slightly exceeding I1 (14.76 t ha^−1^). This indicates that mild deficit irrigation may enhance sugar productivity when a modest increase in sucrose concentration compensates for the slight reduction in root biomass.

The reduction in yield under severe water stress is expected because sugar beet root expansion, canopy duration, and assimilate translocation are highly dependent on adequate soil moisture. Water deficit limits photosynthesis, accelerates senescence, and restricts sink development, resulting in smaller storage roots and lower sugar accumulation. Similar responses have been widely reported in sugar beet and other root crops grown under deficit irrigation (Verma et al., 2025; Ismail et al., 2025).

Importantly, the superior performance of I2 in sugar yield suggests that full irrigation is not always agronomically optimal. Under some environments, a mild deficit can reduce excessive vegetative growth, improve assimilate partitioning, and enhance sugar concentration without major yield penalties. This supports the growing concept of strategic deficit irrigation for maximizing economic water productivity rather than maximizing biomass alone.

### 3.4 Water applied and water-use efficiency under irrigation regimes

**Figure 6:**
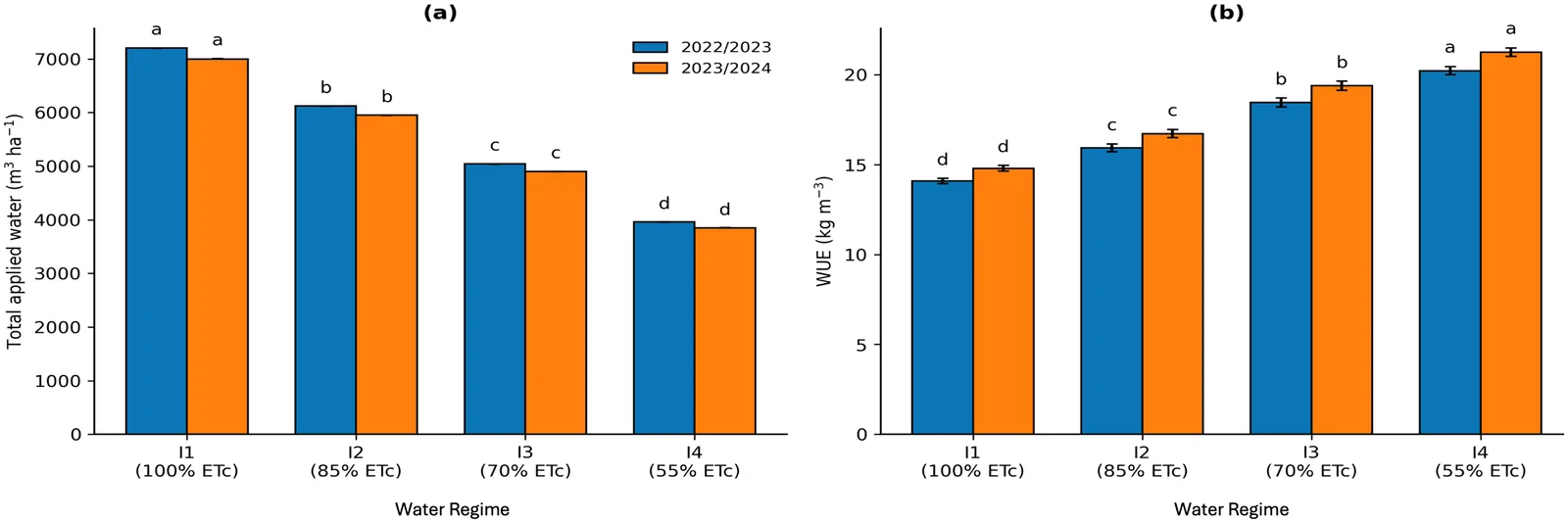
Effect of irrigation regimes on **(a)** total applied water and **(b)** water-use efficiency (WUE) of sugar beet during the 2022/2023 and 2023/2024 seasons. Bars represent mean ± SE. Different letters indicate significant differences at *P* ≤ 0.05.

As expected, total applied water decreased sharply with increasing irrigation deficit (**Figure 6**). Water inputs declined from 7200 and 7000 m^3^ ha^−1^ under I1 to 3960 and 3850 m^3^ ha^−1^ under I4 in the first and second seasons, respectively.

Despite lower yields, WUE increased significantly as irrigation decreased. Values rose from 14.08 to 20.22 kg m^−3^ in 2022/2023 and from 14.79 to 21.25 kg m^−3^ in 2023/2024 from I1 to I4. This demonstrates that severe water restriction improved the efficiency with which irrigation water was converted into economic yield.

The inverse relationship between total yield and WUE is a classical response under deficit irrigation. When irrigation volume is reduced more sharply than yield declines, WUE increases. However, high WUE does not necessarily imply maximum profitability if total sugar production becomes too low. Therefore, irrigation management should target the optimum balance between yield retention and water savings rather than the highest WUE value alone.

Recent irrigation studies consistently support this trade-off, showing that moderate deficits often provide the best compromise between productivity and water conservation (Seleiman et al., 2021; Abdelfattah and Mostafa, 2024; Hajirad et al., 2026).

### 3.5 Root yield, sugar yield, and WUE responses to hydrogel rates

Hydrogel application substantially improved productivity and water-use efficiency (**Table 3**). Root yield increased from 88.12 to 96.39 t ha^−1^ in 2022/2023 and from 90.01 to 98.47 t ha^−1^ in 2023/2024 as hydrogel increased from H0 to H3. Sugar yield increased from 12.65 to 15.52 t ha^−1^ and from 13.21 to 15.15 t ha^−1^ across the same treatments.

**Table 3:** Effect of hydrogel application rates on root yield, sugar yield, total applied water, and water-use efficiency (WUE) of sugar beet during the 2022/2023 and 2023/2024 seasons.

Treatment	2022/2023				2023/2024				
	Root yield (t ha^-1^)	Sugar yield (t ha^-1^)	Total applied water (m^3^ ha^-1^)	WUE (kg m^-3^)		Root yield (t ha^−1^)	Sugar yield (t ha^-1^)	Total applied water (m^3^ ha^−1^)	WUE (kg m^-3^)
H0 (0 kg ha^−1^)	88.12 ± 2.05 ^b^	12.65 ± 0.42 ^b^	5580	16.29 ± 0.62 ^a^		90.01 ± 2.10 ^b^	13.21 ± 0.43 ^b^	5425	17.12 ± 0.65 ^a^
H1 (5 kg ha^−1^)	91.64 ± 2.14 ^ab^	13.48 ± 0.42 ^ab^		16.94 ± 0.64 ^a^		93.61 ± 2.18 ^ab^	14.53 ± 0.44 ^ab^		17.80 ± 0.67 ^a^
H2 (10 kg ha^−1^)	95.70 ± 2.25 ^a^	14.33 ± 0.47 ^a^		17.71 ± 0.69 ^a^		97.76 ± 2.30 ^a^	14.96 ± 0.48 ^a^		18.61 ± 0.72 ^a^
H3 (15 kg ha^−1^)	96.39 ± 2.35 ^a^	15.52 ± 0.48 ^a^		17.82 ± 0.68 ^a^		98.47 ± 2.40 ^a^	15.15 ± 0.50 ^a^		18.72 ± 0.71 ^a^

Data represent mean ± SE. Different letters indicate significant differences at *P* ≤ 0.05.

Similarly, WUE increased from 16.29 to 17.82 kg m^-3^ in the first season and from 17.12 to 18.72 kg m^-3^ in the second season. These gains confirm that hydrogel not only improved productivity but also enhanced the efficiency of water use.

The positive hydrogel effect is attributable to greater soil moisture storage, slower water release, improved root hydration, and enhanced nutrient availability. In sandy soils, these mechanisms are especially valuable because rapid drainage and low field capacity typically intensify crop water stress. Similar improvements in yield and WUE due to hydrogel application have been reported under field and controlled conditions (Abdelghafar et al., 2024; Badr et al., 2024; Bhuyan and Jeong, 2025).

Again, the small difference between H2 and H3 suggests a response plateau, implying that moderate hydrogel rates may be more economically efficient than the highest tested dose.

### 3.6 Interaction effects of irrigation regime × hydrogel rate

The combined analysis of variance revealed that Year, irrigation regime, and hydrogel application significantly affected root yield, sugar yield, and water-use efficiency (WUE) (*P* < 0.001). However, the irrigation × hydrogel interaction was not statistically significant for root yield (F = 0.72, *P* = 0.690), sugar yield (F = 1.04, *P* = 0.412), or WUE (F = 0.75, *P* = 0.664), indicating that the beneficial effects of hydrogel were generally consistent across the different irrigation regimes (**Table 4**).

**Table 4:** Combined ANOVA for root yield, sugar yield and water-use efficiency.

Trait	Source of variation	df	F-value	*P*-value	Significance
Root yield	Year	1	13.33	0.0004	***
	Irrigation (I)	3	291.29	<0.001	***
	Hydrogel (H)	3	50.57	<0.001	***
	I × H	9	0.72	0.690	ns
Sugar yield	Year	1	45.04	<0.001	***
	Irrigation	3	460.63	<0.001	***
	Hydrogel	3	93.42	<0.001	***
	I × H	9	1.04	0.412	ns
Water-use efficiency	Year	1	70.63	<0.001	***
	Irrigation	3	722.30	<0.001	***
	Hydrogel	3	49.06	<0.001	***
	I × H	9	0.75	0.664	ns

ns, not significant (P ≥ 0.05); * significant at P < 0.05; ** significant at P < 0.01; *** significant at P < 0.001 according to LSD test.

**Figure 7:**
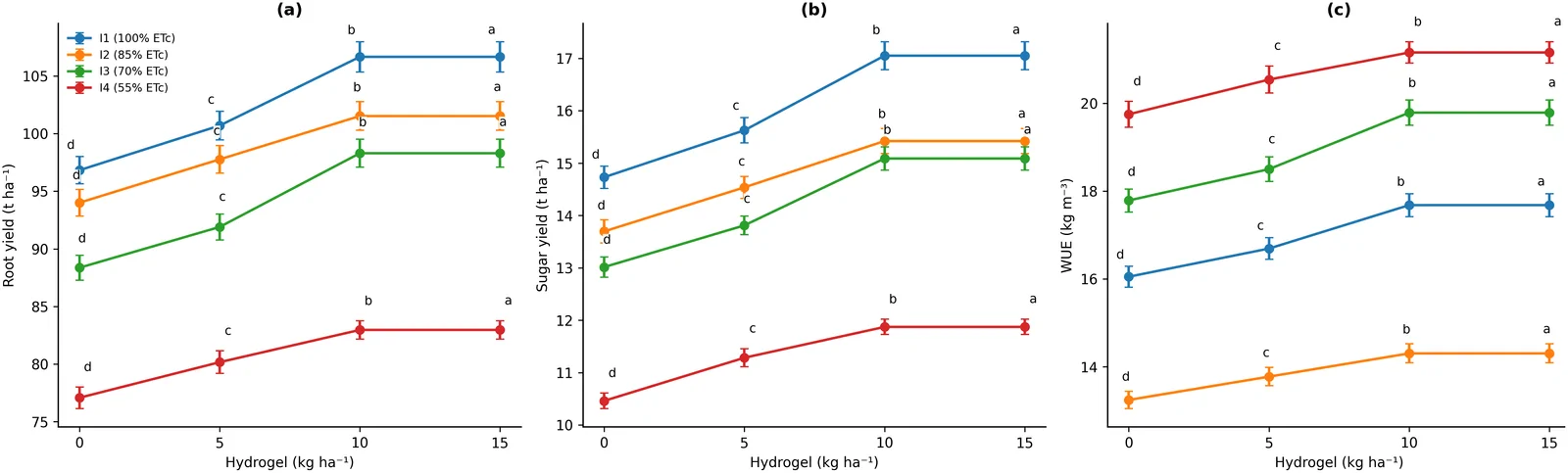
Interaction effects of irrigation regimes and hydrogel rates on root yield, sugar yield, and water-use efficiency (WUE) of sugar beet during the two growing seasons. Values are treatment means ± SE. Different letters indicate significant differences at *P* ≤ 0.05.

Despite the absence of a significant interaction, the treatment combinations exhibited distinct numerical responses (**Figure 7**). Hydrogel application consistently enhanced root yield, sugar yield, and WUE under all irrigation regimes, with the magnitude of improvement increasing as water availability declined. Compared with the untreated control (H0), hydrogel application increased root yield by 4.2-9.3% under full irrigation (I1), whereas the improvement increased to 9.7-16.3% under severe deficit irrigation (I4). Similarly, sugar yield increased by 3.5-8.1% under full irrigation and by 8.9-14.8% under severe deficit irrigation, while WUE improved by 3.1-6.3% and 6.5-10.8%, respectively. These findings indicate that although hydrogel exerted a consistent positive effect across irrigation regimes, its agronomic benefits became progressively greater under water-deficit conditions.

As illustrated in **Figure 7**, the highest root and sugar yields were generally achieved under I1 or I2 combined with H2 or H3, whereas the greatest WUE values were recorded under I4 combined with H2 or H3. These results indicate that hydrogel application did not completely offset the yield reduction associated with severe water deficit but substantially enhanced crop productivity and water-use efficiency under limited irrigation. The greater relative improvements observed under progressive water deficit suggest that hydrogel mitigated drought stress by increasing soil water retention and improving water availability within the root zone, thereby sustaining crop productivity under moisture-limited conditions.

**Figure 8:**
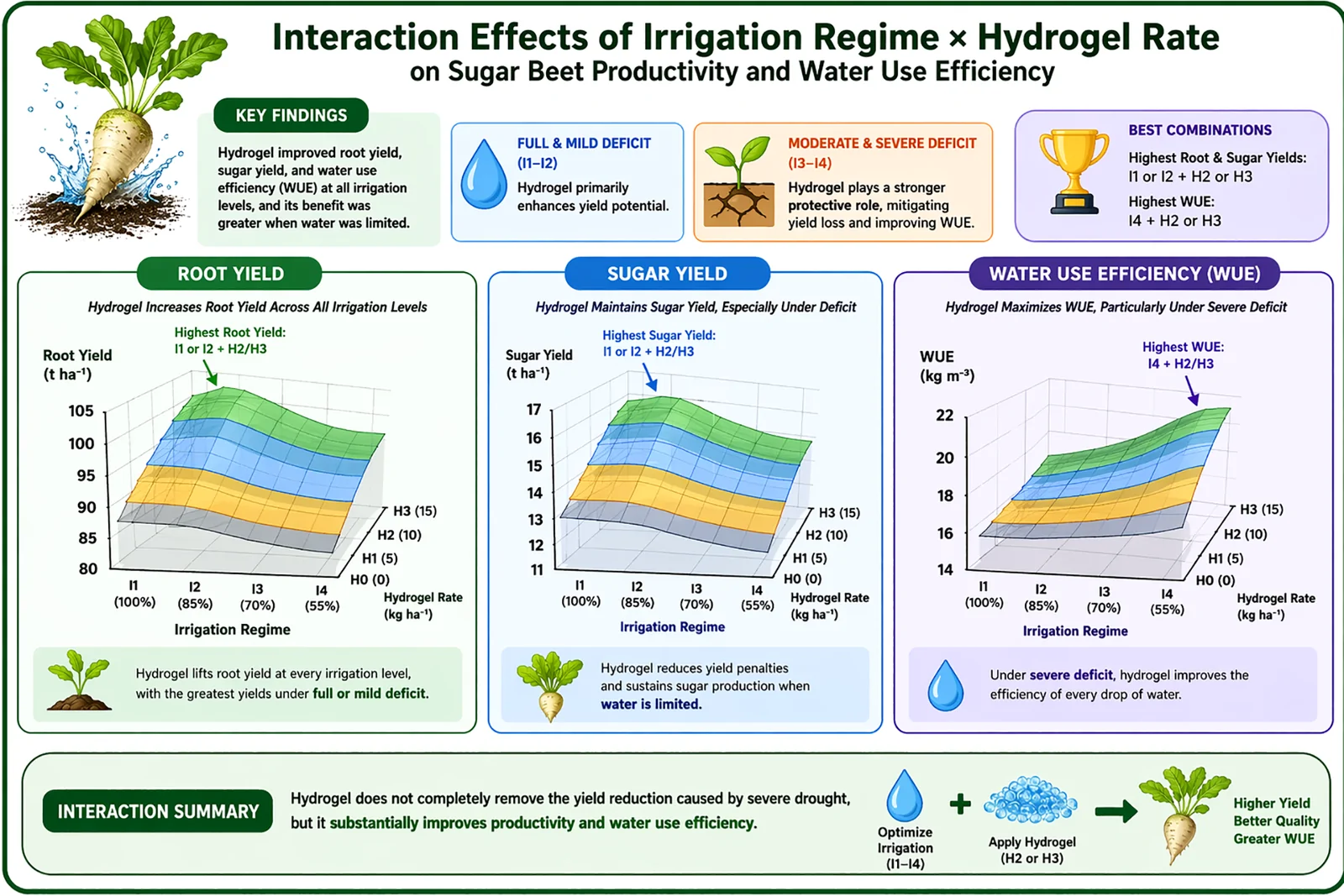
Graphical abstract illustrating the interactive effects of irrigation regime and hydrogel rate on sugar beet root yield, sugar yield, and water-use efficiency under sandy soil conditions.

From a practical perspective, these findings demonstrate that hydrogel application is most effective when integrated with optimized irrigation management rather than used as a standalone soil amendment. The consistent positive response across all irrigation regimes, together with the greater relative improvements observed under deficit irrigation, supports the use of hydrogels as an effective strategy for enhancing crop resilience, improving water-use efficiency, and sustaining sugar beet productivity in sandy soils under water-limited conditions. This interpretation is further illustrated in the Graphical Abstract (**Figure 8**), which summarizes the complementary roles of optimized irrigation scheduling and hydrogel application in improving crop productivity and water-use efficiency. These findings are consistent with recent climate-smart agricultural strategies advocating the integration of deficit irrigation and soil-conditioning amendments to improve resource-use efficiency and stabilize crop production in arid and semi-arid environments (Ismail et al., 2025; Hajirad et al., 2026).

### 3.7 Economic analysis

Economic analysis (**Table 5**) revealed considerable variation in profitability among the irrigation regime × hydrogel treatment combinations. Gross return generally increased with hydrogel application owing to the corresponding increase in root yield. However, this increase in revenue was accompanied by higher production costs due to the additional expense of hydrogel application. Consequently, the economic benefit of higher hydrogel rates depended on the balance between yield improvement and additional input costs. The highest gross return (126,120 EGP ha^-1^) and net return (112,320 EGP ha^-1^) were obtained under the I1H3 treatment (100% ETc combined with 15 kg ha^-1^ hydrogel), reflecting the superior root productivity achieved under this treatment. In contrast, I4H0 (55% ETc without hydrogel) recorded the lowest gross return because severe water deficit substantially reduced root yield. These findings demonstrate that maximizing crop productivity remains the primary driver of gross income, particularly under adequate irrigation conditions.

**Table 5:** Economic performance of irrigation × hydrogel treatments based on combined-season estimated means.

Irrigation	Hydrogel	Root yield (t ha^-1^)	Water applied (m^3^ ha^-1^)	Gross return (EGP ha^-1^)	Total cost (EGP ha^-1^)	Net return (EGP ha^-1^)	BCR	EWP (EGP m^-3^)
I1	H0	97.57	7,100	117,080	12,550	104,530	9.33	14.72
	H1	101.13	7,100	121,352	13,300	108,052	9.12	15.22
	H2	105.23	7,100	126,278	14,050	112,228	8.99	15.81
	H3	105.93	7,100	127,118	14,800	112,318	8.59	15.82
I2	H0	93.57	6,035	112,286	12,018	100,269	9.34	16.61
	H1	97.13	6,035	116,558	12,768	103,791	9.13	17.20
	H2	101.24	6,035	121,484	13,518	107,967	8.99	17.89
	H3	101.94	6,035	122,324	14,268	108,057	8.57	17.91
I3	H0	89.09	4,970	106,904	11,485	95,419	9.31	19.20
	H1	92.65	4,970	111,176	12,235	98,941	9.09	19.91
	H2	96.75	4,970	116,102	12,985	103,117	8.94	20.75
	H3	97.45	4,970	116,942	13,735	103,207	8.51	20.77
I4	H0	76.02	3,905	91,220	10,952	80,268	8.33	20.56
	H1	79.58	3,905	95,492	11,702	83,790	8.16	21.46
	H2	83.68	3,905	100,418	12,452	87,966	8.06	22.53
	H3	84.38	3,905	101,258	13,202	88,056	7.67	22.55

Gross return was calculated from root yield at 1,200 EGP t^-1^. Total cost included a base production cost of 9,000 EGP ha^-1^, irrigation water at 0.50 EGP m^-3^, and hydrogel at 150 EGP kg^-1^. BCR, benefit-cost ratio; EWP, economic water productivity.

The benefit-cost ratio (BCR) varied from 8.52 to 9.34, with the highest value recorded under I2H0. This result indicates that moderate deficit irrigation without hydrogel generated the greatest economic return relative to production costs. Although increasing hydrogel application consistently improved root yield, the associated increase in input costs reduced the incremental economic advantage of the highest hydrogel application rate. This finding suggests that maximizing biological productivity does not necessarily maximize economic efficiency, emphasizing the importance of balancing production costs with yield gains. Economic water productivity (EWP) increased progressively as irrigation water application decreased, reaching its highest value under I4H3. This demonstrates that combining hydrogel application with deficit irrigation substantially increased the economic return generated per unit of irrigation water applied. Such an improvement is particularly important under arid and semi-arid conditions, where irrigation water represents a major limiting resource and production systems increasingly aim to maximize economic returns from limited water supplies rather than absolute crop yield.

### 3.8 Agronomic implications and concluding interpretation

Collectively, the results reveal three central outcomes. First, severe irrigation deficit improved sucrose concentration and WUE but significantly reduced root and sugar yield. Second, hydrogel application consistently enhanced quality, yield, and WUE by improving soil moisture conditions and reducing impurity accumulation. Third, the most balanced agronomic response was achieved when moderate irrigation deficit was combined with intermediate-to-high hydrogel rates.

Accordingly, treatments such as I2 or I3 integrated with H2 or H3 appear more suitable than either full irrigation without amendment or severe water deficit alone. These combinations conserved water while sustaining high sugar productivity and acceptable processing quality. For sandy soils under arid and semi-arid environments, such integrated management may represent a realistic and scalable strategy for sustainable sugar beet production under increasing water scarcity.

## 4 Conclusion

Irrigation regime and hydrogel application significantly influenced sugar beet productivity, quality, and WUE under sandy soil conditions. Severe water deficit improved sucrose concentration and WUE, but markedly reduced root and sugar yields. In contrast, hydrogel application enhanced sugar recovery, reduced impurities, and improved root and sugar yields as well as water use efficiency through better soil moisture retention.

Hydrogel was beneficial under all irrigation levels, with greater advantages under water-deficit conditions. The highest root and sugar yields were obtained under I1-I2 combined with H2-H3, while the highest WUE was achieved under I4 with H2 or H3.

Therefore, applying moderate deficit irrigation (85-70% ETc) together with 10–15 kg ha^−1^ hydrogel is recommended as an efficient and sustainable strategy for sugar beet production in arid sandy soils, enabling substantial water savings with minimal yield reduction.

Overall, integrating economic analysis with agronomic evaluation demonstrated that the optimum management strategy should be selected based not only on crop productivity but also on production costs and water-use efficiency. Accordingly, hydrogel application combined with optimized irrigation management offers a practical and economically viable approach for enhancing the sustainability and profitability of sugar beet production in arid and semi-arid sandy soils.

## Data Availability

The original contributions presented in the study are included in the article/supplementary material. Further inquiries can be directed to the corresponding author.
